# Using group based trajectory modeling for assessing medication adherence to nintedanib among idiopathic pulmonary fibrosis patients

**DOI:** 10.1186/s12890-023-02496-3

**Published:** 2023-06-27

**Authors:** Mona Nili, Andrew J. Epstein, Dominic Nunag, Amy Olson, Bijan Borah

**Affiliations:** 1 Boehringer Ingelheim Pharmaceuticals, Inc, Ridgefield, CT USA; 2 Medicus Economics, LLC, Milton, MA USA; 3 Mayo Clinic, Rochester, MN USA

**Keywords:** Idiopathic pulmonary fibrosis, Medication adherence, Nintedanib, Group based trajectroy modeling, Real world data

## Abstract

**Background and objective:**

Adherence to antifibrotic medications has been evaluated in a few studies using annual proportion of days covered (PDC), a common adherence metric. However, PDC alone cannot identify and distinguish between different patterns of adherence over time, which can be accomplished using group-based trajectory models (GBTM) of monthly PDC. The objective is to assess nintedanib adherence trajectories using GBTM and identify characteristics of patients within each trajectory group.

**Methods:**

Individuals with idiopathic pulmonary fibrosis (IPF) who initiated nintedanib during 10/1/2014–12/31/2018 were identified in 100% Medicare claims and enrollment data. The sample consisted of community-dwelling older adults (≥ 66 years) with continuous coverage in Medicare Parts A, B and D for one year before (baseline) and after (follow-up) initiating nintedanib. A series of GBTMs of adherence was estimated to identify the best-fitting specification. Patients were then grouped based on their estimated adherence trajectories. Associations between baseline patient characteristics, including demographics, comorbidities, and health care use, and group membership probabilities were quantified as odds ratios using fractional multinomial logit modeling.

**Results:**

Among the 1,798 patients initiating nintedanib, mean age was 75.4 years, 61.1% were male, and 91.1% were non-Hispanic white. The best-fitting GBTM had five adherence trajectory groups: high adherence (43.1%), moderate adherence (11.9%), high-then-poor adherence (10.4%), delayed-poor adherence (13.2%), and early-poor adherence (21.5%). The principal factors associated with higher odds of being in at least one of the poor-adherence groups were older age, female sex, race and ethnicity other than non-Hispanic white, and number of medications during baseline.

**Conclusions:**

GBTM identified distinct patterns of nintedanib adherence for the IPF patient cohort. Identifying adherence trajectory groups and understanding the characteristics of their members provide more actionable information to personalize interventions than conventional metrics of medication adherence.

**Supplementary Information:**

The online version contains supplementary material available at 10.1186/s12890-023-02496-3.

## Introduction

Idiopathic pulmonary fibrosis (IPF) is a rare interstitial lung disease that is accompanied by a chronic progressive-fibrosing condition and typically presents among older adults. [1–5] For treatment of IPF, nintedanib, an antifibrotic medication, can be used to slow progression (as measured by forced vital capacity reduction) in patients with less-advanced disease [6–8] and in patients with more-advanced disease. [9–11] Nintedanib use has also been shown to reduce mortality risk. [12–14] A key determinant of nintedanib effectiveness is being adherent to the recommended dose. Adherence to nintedanib has been evaluated in a few recent studies in the United States. [15–17] These studies used PDC to measure adherence either as average PDC or percentage of patients with PDC ≥ 80%.

There are several approaches to measuring adherence, each with its own strengths and limitations. [18] A common method in real-world settings involving prescription drug claims databases, is the proportion of days covered (PDC), which is defined for a specific medication as the number of days supplied divided by the total number of days in the study period. [19] Although PDC is useful in that it offers in a single number a metric of medication adherence over a predefined period, its simplicity masks any heterogeneity in adherence between patients and specifically it does not capture changes in individual patient adherence over time.

An alternative approach is to use group-based trajectory modeling (GBTM) to detect trajectories of medication adherence over time and identify clusters of individuals who follow similar trajectories or longitudinal patterns of adherence. [20] GBTM has been used to understand adherence to medications in several therapeutic areas. [21] Longitudinal evaluation of medication adherence using GBTM can yield more informative classifications compared to traditional methods such as PDC. [22].

Given the limitations of PDC and the importance of understanding adherence to nintedanib, the objective of this study was to assess nintedanib adherence trajectories using GBTMs and identify characteristics of patients following each of these trajectories.

## Methods

### Study design and data source

This was a non-interventional cohort study using existing administrative data. The study used 100% enrollment and claims data from the U.S. Medicare program covering 2013 through 2019. The enrollment file contains monthly information on individuals’ enrollment in each part of Medicare, demographic information, residential location, and date of death. Claims were available for inpatient hospital, skilled nursing, and outpatient facility services (Part A), physician and other professional services (Part B), and outpatient prescription drugs (Part D). Part D claims include standardized prescription-level information, including drug names, National Drug Codes, strength, quantity, days’ supply, and fill date. This study was determined to be exempt from review by the Western Copernicus Group (WCG) Institutional Review Board.

### Study cohort

**Fig. 1:**
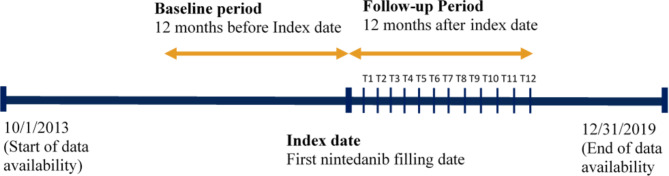
Study design schematic

The study cohort consisted of community-dwelling older adults (≥ 66 years old) with IPF who initiated treatment with nintedanib between 10/01/2014 and 12/31/2018. The index date was defined as the date of the first prescription fill for nintedanib. A study design schematic is displayed in Fig. 1. Patients were required to have continuous coverage in Medicare Parts A, B and D for 12 months prior to their index date (baseline period) to preclude prior antifibrotic use and for 12 months following index date (follow-up period) to enable complete adherence follow-up. Additional sample inclusion criteria included: qualifying for Medicare based on age; having at least one inpatient or two outpatient claims (≥ 14 days apart) with an IPF diagnosis code (ICD-10-CM: J84.112; ICD-9-CM: 516.31) during the baseline period; not having any antifibrotic use during the baseline period; not having any of the following conditions: a lung transplant, use of skilled nursing facilities, use of long-term care facilities or hospice care, and dual-eligibility for Medicaid and Medicare during the baseline and follow-up periods; not having any claims with a diagnosis code for lung cancer or autoimmune conditions during the baseline period; not having any use of pirfenidone, another antifibrotic, that overlapped with nintedanib use during the follow-up period; and not having a death date before the end of the follow-up period.

### Outcome measures

Part D claims for nintedanib were used to construct monthly supply calendars based on each prescription claim’s service date and days’ supply, while accounting for possible stockpiling from overlapping supply from multiple claims. For each 30-day month following the nintedanib initiation date, PDC was calculated from the supply calendar as the sum of days with supply divided by 30. Monthly adherence to nintedanib was defined by dichotomizing PDC, where monthly PDC ≥ 80% indicated adherence. [22, 24] The 12 monthly dichotomous PDCs were then used in building the GBTMs.

### Other measures

To identify differences between adherence trajectory groups, patient demographic and socioeconomic characteristics, comorbid conditions, and health care use were assessed on the index date and during the 12-month baseline period. Demographic and socioeconomic characteristics included patient age at index date, sex, race and ethnicity, Census region, the Social Deprivation Index and calendar year of index date. The Social Deprivation Index is a measure combining ZIP Code-level Census data on percent living in poverty, percent with less than 12 years of education, percent of single-parent household, percent living in rented housing unit, percent living in overcrowded housing unit, percent of households without a car, and percent of non-employed adults under 65 years of age. [25] It ranges from 0 to 100, with higher values indicating more social deprivation.

To account for general disease burden, Gagne’s comorbidity index was used. This comorbidity index includes 20 comorbid conditions (i.e. metastatic cancer, congestive heart failure, dementia, renal failure, weight loss, hemiplegia, alcohol abuse, any tumor, cardiac arrhythmias, chronic pulmonary disease, coagulopathy, complicated diabetes, deficiency anemia, fluid and electrolyte disorders, liver disease, peripheral vascular disease, psychosis, pulmonary circulation disorders, HIV/AIDS, and hypertension). [26, 27] Separate indicators were constructed for the combination of Alzheimer’s disease and dementia, which we hypothesized might affect patients’ ability to adhere to medication, and for the following IPF-related conditions: congestive heart failure, asthma, chronic obstructive pulmonary disease (COPD)/ emphysema, gastroesophageal reflux disease, hypoxia, pulmonary hypertension, and sleep apnea. IPF-related medical service receipt included indicators for high-resolution computed tomography (HRCT), lung biopsy, and oxygen therapy/supplemental oxygen.

Baseline general health care use included the count of unique medications (based on the generic medication name in the Part D outpatient pharmacy claims fields), the total out-of-pocket spending by the patient on all medical and pharmacy claims that started during the baseline period (adjusted to 2019 dollars using the Medical Care component of the US Consumer Price Index), an indicator for whether the patient had a baseline inpatient hospitalization, an indicator for whether the prescriber of the index nintedanib prescription had a pulmonology specialty, and an indicator for whether the patient had a baseline claim from at least one clinician with a pulmonology specialty.

### Analysis

GBTM was used to identify the best-fitting collection of trajectory groups to explain nintedanib adherence for the 12 months after initiation. A GBTM takes as prespecified inputs the number of distinct groups assumed to be in the sample and, for each group, the order of the polynomial function of elapsed time since nintedanib initiation describing the trajectory of that group. A GBTM consists of multiple simultaneously estimated regression models, which in this application included a multinomial logit model of adherence group membership. For each adherence group, a separate binary logit model of the adherence (PDC ≥ 80%) indicators was used as a polynomial function of month.

Convention in building GBTMs is to first determine the best-fitting number of groups and then determine the best-fitting polynomial order for each group. [28, 29] Thirty model specifications were considered initially as formed by varying the number of assumed groups between 1, 2, 3, 4 and 5 while varying the assumed polynomial order for all adherence trajectory groups in that specification between zero, linear, quadratic, cubic, quartic, and quintic. The model specification with the best Bayesian Information Criterion (BIC) value was deemed to yield the optimal number of groups as well as the maximum polynomial order for any group. Following identification of the number of groups and maximum polynomial order, the order of each group was reduced from the maximum by one for any group for which the coefficient on the highest term in that group’s polynomial was not statistically significantly different from zero (and the model was re-estimated) until the coefficient on each group’s highest term was statistically significant and the smallest group had ≥ 5% of the sample assigned to it. [28, 30]

Model goodness-of-fit was assessed several ways. The calibration between the estimated adherence trajectories and observed adherence was evaluated by checking that the sample-wide mean posterior probabilities of group membership were ≥ 0.7 for all groups and that the odds of correct classification were > 5 for all groups. The relative entropy value, a model-wide measure of confidence in group classification bound between 0 and 1 (with higher certainty closer to 1), is recommended to be at least 0.7. [28, 30] Spaghetti plots of individual adherence patterns for each adherence trajectory group were visually inspected to gauge how well the individual patterns matched the estimated group trajectory. [29].

Descriptive statistics of the baseline characteristics were calculated stratified by patients’ adherence trajectory group membership. For unadjusted analysis, ANOVA was used for continuous measures and χ^2^was used for categorical measures. For adjusted analysis, a regression model was estimated to assess independent associations between baseline patient characteristics and the GBTM-derived predicted probability of belonging to a specific adherence trajectory group. Because the GBTM yielded for each patient a set of predicted probabilities of belonging to each group, the model was specified as fractional multinomial logit and odds ratios were used to quantify adjusted associations. [31] Model standard errors were made robust to heteroskedasticity of unknown form. Statistical significance was based on 2-sided tests with α = 0.05. All data management and analyses were conducted with SAS 9.4 (SAS Institute Inc; Cary, NC) and Stata 17.0 software (StataCorp; College Station, TX).

### Patient and public involvement

Patients or the public were not involved in the design, or conduct, or reporting, or dissemination plans of our study.

## Results

**Fig. 2:**
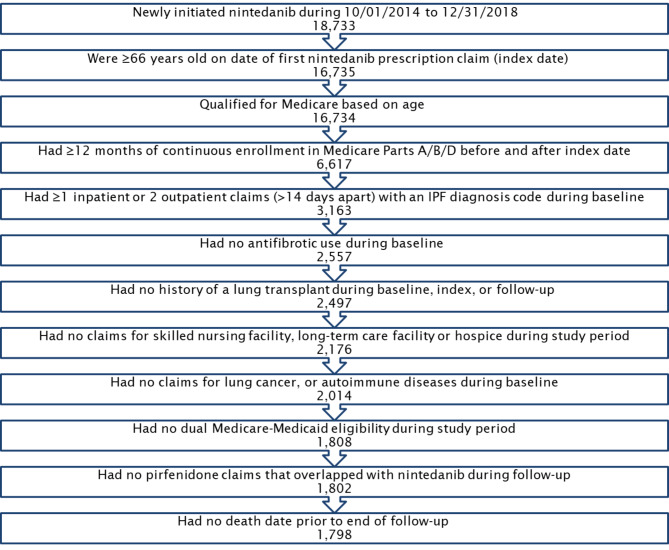
Sample selection criteria

There were 18,733 Medicare beneficiaries with at least one prescription claim for nintedanib between 10/01/2014 and 12/31/2018. After sample selection criteria were applied, the final analytic sample consisted of 1,798 patients with at least one prescription claim for nintedanib (Fig. 2).

Sample characteristics are shown in Table 1. Mean age was 75.4 years; 61.1% were male; 91.1% were non-Hispanic white; and mean overall PDC during the 12 months of follow-up was 0.653. The percentage of patients with PDC ≥ 80% was 51.2%. The mean count of 20 general comorbid conditions during the 1-year baseline was 3.9. Among the IPF-related comorbidities and services, about half of the sample had claims during the baseline for gastroesophageal reflux disease (51.3%) and COPD/ emphysema (48.7%), and three-quarters (76.6%) received a high-resolution CT scan.

**Table 1:** Baseline patient characteristics overall and by trajectory group

	Total	High adherence	Moderate adherence	High-then-poor adherence	Delayed-poor adherence	Early-poor adherence	P-value
Sample size, n (%)	1,798 (100.0)	781 (43.4)	202 (11.2)	190 (10.6)	255 (14.2)	370 (20.6)	
**Demographic characteristics**							
Age (yrs), mean (sd)	75.4 (5.5)	74.7 (5.4)	75.2 (5.4)	75.9 (5.6)	76.3 (5.6)	76.2 (5.6)	< 0.001
Female, n (%)							< 0.001
Male	1,098 (61.1)	530 (67.9)	113 (55.9)	124 (65.3)	138 (54.1)	193 (52.2)	
Female	700 (38.9)	251 (32.1)	89 (44.1)	66 (34.7)	117 (45.9)	177 (47.8)	
Race/ethnicity other than non-Hispanic White, n (%)							0.37
Non-Hispanic White	1,638 (91.1)	718 (91.9)	183 (90.6)	171 (90.0)	237 (92.9)	329 (88.9)	
Not non-Hispanic White	160 (8.9)	63 (8.1)	19 (9.4)	19 (10.0)	18 (7.1)	41 (11.1)	
Census region, n (%)							0.48
Northeast	256 (14.2)	104 (13.3)	31 (15.3)	27 (14.2)	48 (18.8)	46 (12.4)	
Midwest	416 (23.1)	172 (22.0)	46 (22.8)	51 (26.8)	55 (21.6)	92 (24.9)	
South + Puerto Rico	791 (44.0)	344 (44.0)	84 (41.6)	79 (41.6)	113 (44.3)	171 (46.2)	
West	335 (18.6)	161 (20.6)	41 (20.3)	33 (17.4)	39 (15.3)	61 (16.5)	
Social Deprivation Index, mean (sd)	39.9 (26.0)	40.9 (26.0)	38.3 (25.5)	38.6 (26.4)	39.3 (25.9)	40.0 (25.9)	0.64
**Clinical characteristics**							
Gagne comorbidity count, mean (sd)	3.9 (2.2)	3.8 (2.2)	3.8 (2.2)	3.8 (2.2)	4.0 (2.2)	4.1 (2.3)	0.15
Congestive heart failure, n (%)	616 (34.3)	257 (32.9)	65 (32.2)	53 (27.9)	101 (39.6)	140 (37.8)	0.04
Asthma, n (%)	296 (16.5)	132 (16.9)	34 (16.8)	31 (16.3)	32 (12.5)	67 (18.1)	0.45
Chronic obstructive pulmonary disease/ emphysema, n (%)	843 (46.9)	373 (47.8)	86 (42.6)	86 (45.3)	115 (45.1)	183 (49.5)	0.52
Gastroesophageal reflux disease, n (%)	923 (51.3)	398 (51.0)	104 (51.5)	97 (51.1)	125 (49.0)	199 (53.8)	0.83
Hypoxia, n (%)	537 (29.9)	234 (30.0)	57 (28.2)	63 (33.2)	83 (32.5)	100 (27.0)	0.47
Pulmonary hypertension, n (%)	356 (19.8)	156 (20.0)	36 (17.8)	39 (20.5)	48 (18.8)	77 (20.8)	0.92
Sleep apnea, n (%)	529 (29.4)	243 (31.1)	52 (25.7)	57 (30.0)	62 (24.3)	115 (31.1)	0.19
**IPF-related medical services**							
High-resolution CT scan, n (%)	1,378 (76.6)	597 (76.4)	157 (77.7)	144 (75.8)	200 (78.4)	280 (75.7)	0.93
Lung biopsy, n (%)	296 (16.5)	134 (17.2)	37 (18.3)	26 (13.7)	35 (13.7)	64 (17.3)	0.49
Oxygen therapy or supplemental oxygen, n (%)	302 (16.8)	128 (16.4)	28 (13.9)	32 (16.8)	45 (17.6)	69 (18.6)	0.67
**Medical care use**							
Number of medications, mean (sd)	11.4 (5.6)	10.9 (5.5)	11.4 (5.9)	11.6 (5.6)	12.0 (5.7)	11.8 (5.4)	0.03
Any inpatient hospitalization, n (%)	597 (33.2)	269 (34.4)	61 (30.2)	60 (31.6)	71 (27.8)	136 (36.8)	0.14
Any emergency department use, n (%)	729 (40.5)	293 (37.5)	77 (38.1)	82 (43.2)	105 (41.2)	172 (46.5)	0.050
**Outpatient pharmacy spending**							
Pharmacy spending - total, mean (sd)	3,550 (9,112)	3,670 (10,951)	3,042 (4,718)	2,899 (4,078)	3,711 (8,047)	3,796 (9,229)	0.72
Pharmacy spending - OOP, mean (sd)	807 (983)	798 (1,062)	732 (734)	759 (801)	807 (971)	891 (1,019)	0.35
**All-cause medical care spending**							
Medical spending - total, mean (sd)	17,021 (20,164)	17,634 (23,297)	16,280 (18,712)	16,029 (15,873)	15,656 (16,455)	17,581 (17,982)	0.57
Medical spending - OOP, mean (sd)	2,737 (2,531)	2,724 (2,745)	2,635 (2,161)	2,702 (2,172)	2,645 (2,244)	2,900 (2,608)	0.69
**Pulmonology**							
Index nintedanib prescriber was pulmonologist, n (%)	1,337 (74.4)	584 (74.8)	150 (74.3)	140 (73.7)	188 (73.7)	275 (74.3)	1.00
Any pulmonologist visits during baseline, n (%)	1,679 (93.4)	730 (93.5)	187 (92.6)	172 (90.5)	244 (95.7)	346 (93.5)	0.29

Note:

1) Patients were assigned to the adherence trajectory with the highest GBTM-derived predicted probability of membership

2) Significant group differences in nintedanib adherence trajectories were based on ANOVA for continuous variable and chi-square tests for categorical variables. Percentages may not sum to 100% due to rounding

3) 28 cases were missing data on Social Deprivation Index

4) CT: Computed Tomography

5) OOP: out-of-pocket

6) sd: standard deviation

During the baseline period, nintedanib users had claims for on average 11.4 unique (as identified by generic name) medications. 33% had at least one inpatient admission. Total spending averaged $20,570, split between medical spending ($17,021) and pharmacy ($3,550), while mean total out-of-pocket spending was $3,554.

Mean monthly nintedanib PDC declined from 100% in the month immediately following initiation (which occurred because nintedanib prescriptions come with at least 30 days’ supply) to 77% in month 2 and then monotonically to 51% in month 12 (**Appendix** Fig. 1).

**Fig. 3:**
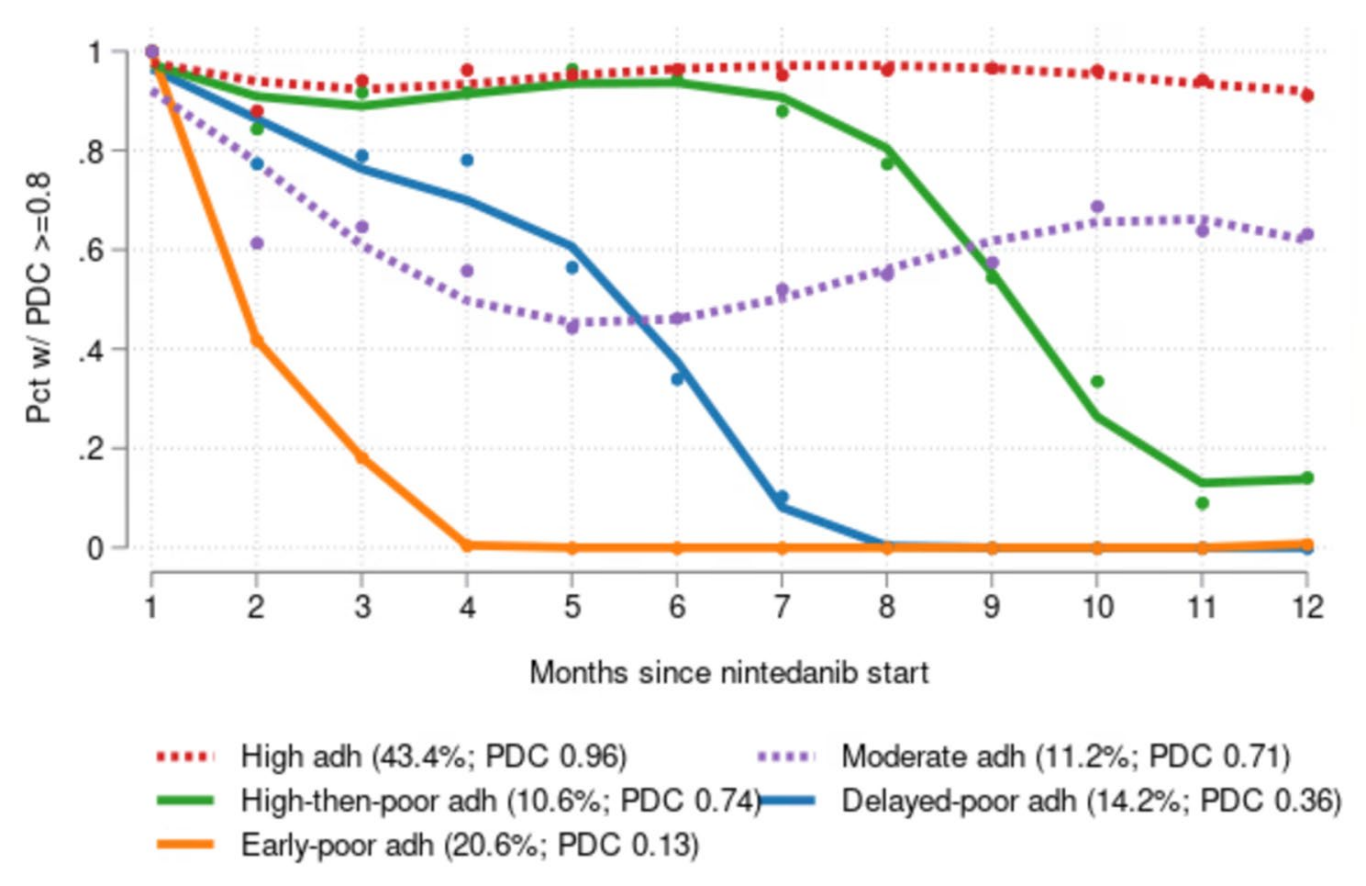
Nintedanib adherence trajectories among Medicare beneficiaries with IPF

The final GBTM specification had five adherence trajectories with polynomial orders of 3, 4, 4, 4, and 3 (Fig. 3). The trajectory plots suggest 5 distinct patterns of nintedanib adherence:

- 43.1% of patients were in the *high adherence* trajectory, with almost 100% share of person-months with PDC ≥ 80% throughout the follow-up period (average PDC: 0.96).
- 11.9% were in the *moderate adherence* trajectory, with a share of person-months with PDC ≥ 80% between roughly 40% and 60% by the third month after nintedanib initiation (average PDC: 0.71).
- 10.4% were in the *high-then-poor adherence* trajectory with adherence that was consistently high until the seventh month before leveling-off in the 11th month (average PDC: 0.74).
- 13.2% were in the *delayed-poor adherence* trajectory, with steadily decreasing adherence until halfway through the follow-up period at which point adherence dropped sharply (average PDC: 0.36).
- 21.5% were in the *early-poor adherence* trajectory, who stopped filling nintedanib prescriptions by the fourth month (average PDC: 0.13).

The GBTM specification satisfied the suggested fitness criteria. The smallest mean posterior probability of group membership for any group was 0.845, which is larger than suggested minimum of 0.7; the smallest odds of correct classification for any group was 29.8, well above the suggested minimum of 5; and the estimated model entropy value (0.836) exceeded the recommended threshold of 0.7. [28, 30] Moreover, spaghetti plots show consistency between patients’ observed adherence patterns and the estimated trajectory of their most-likely adherence trajectory (i.e., the one with the highest posterior probability of group membership as generated by the GBTM) (**Appendix Fig. 2**).

Some baseline patient characteristics varied across adherence trajectories (after assigning each patient to the group for which they had the highest posterior probability of membership), as shown in Table 1. For example, patients in the “delayed-poor adherence” and “early-poor adherence” trajectories were nominally older on average and more frequently female than those in the “high adherence” trajectory.

Adjusted odds ratios from the fractional multinomial logit model of adherence trajectory group membership are presented in Table 2. Relative to membership in the “high adherence” trajectory, older age was associated with higher odds of membership in the “high-then-poor adherence” (adjusted odds ratio [AOR] 1.04, 95% confidence interval [CI]: 1.01, 1.07), “delayed-poor adherence” (AOR: 1.06, 95% CI: 1.03, 1.08), and “early-poor adherence” (AOR: 1.05, 95% CI: 1.03, 1.08) trajectories. Female sex was associated with higher odds of membership in the “moderate adherence” (OR 1.58, 95% CI: 1.19, 2.09), “delayed-poor adherence” (AOR: 1.82, 95% CI: 1.35, 2.43), and “early-poor adherence” (AOR: 2.08, 95% CI: 1.61, 2.69) trajectories. Patients whose race and ethnicity were characterized as other than non-Hispanic white (including missing values) had higher odds of being in the “early-poor adherence” trajectory (AOR: 1.56, 95% CI: 1.02, 2.39). Taking an additional medication during the baseline period was associated with higher odds of being in the “high-then-poor adherence” (AOR: 1.03, 95% CI: 1.00, 1.07) and “delayed-poor adherence” (AOR: 1.05, 95% CI: 1.02, 1.09) trajectories. Having at least one inpatient hospitalization during the baseline period was associated with lower odds of being in the delayed-poor adherence trajectory (AOR: 0.57, 95% CI: 0.38, 0.85).

**Table 2:** Adjusted odds ratios of trajectory group membership relative to high adherence group

	Moderate adherence			High-then-poor adherence			Delayed-poor adherence			Early-poor adherence		
Variables	AOR	95% CI	P	AOR	95% CI	P	AOR	95% CI	P	AOR	95% CI	P
**Demographics**												
Age (yrs)	1.02	(0.99–1.04)	0.17	1.04	(1.01–1.07)	0.007	1.06	(1.03–1.08)	< 0.001	1.05	(1.03–1.08)	< 0.001
Female	1.58	(1.19–2.09)	0.001	1.21	(0.89–1.63)	0.22	1.82	(1.35–2.43)	< 0.001	2.08	(1.61–2.69)	< 0.001
Race/ethnicity other than non-Hispanic White	1.26	(0.78–2.04)	0.34	1.19	(0.71–2.00)	0.52	1.10	(0.64–1.89)	0.72	1.56	(1.02–2.39)	0.04
Census region												
Northeast	ref			ref			ref			ref		
Midwest	0.93	(0.59–1.46)	0.75	1.17	(0.73–1.87)	0.53	0.67	(0.42–1.05)	0.08	1.21	(0.79–1.84)	0.38
South	0.81	(0.53–1.24)	0.33	0.91	(0.58–1.42)	0.68	0.69	(0.46–1.03)	0.07	1.13	(0.76–1.66)	0.55
West	0.83	(0.52–1.33)	0.45	0.70	(0.42–1.17)	0.17	0.51	(0.31–0.83)	0.006	0.79	(0.50–1.23)	0.29
Social Deprivation Index	1.00	(0.99–1.00)	0.20	1.00	(0.99–1.00)	0.25	1.00	(0.99–1.00)	0.44	1.00	(0.99–1.00)	0.12
**Comorbidities**												
Gagne comorbidity count	1.04	(0.95–1.13)	0.38	1.01	(0.92–1.11)	0.85	1.00	(0.90–1.10)	0.93	1.04	(0.96–1.13)	0.38
Alzheimer’s and dementia	0.49	(0.15–1.56)	0.23	0.56	(0.19–1.65)	0.29	1.24	(0.54–2.87)	0.61	0.92	(0.44–1.90)	0.82
Congestive heart failure	0.96	(0.68–1.35)	0.80	0.71	(0.50–1.00)	0.052	1.32	(0.93–1.87)	0.12	1.13	(0.83–1.53)	0.43
Asthma	0.97	(0.69–1.38)	0.88	0.91	(0.62–1.34)	0.64	0.68	(0.45–1.02)	0.06	1.02	(0.73–1.41)	0.91
Chronic obstructive pulmonary disease/ emphysema	0.81	(0.61–1.06)	0.13	0.91	(0.68–1.23)	0.55	0.84	(0.63–1.13)	0.26	0.98	(0.76–1.27)	0.88
Gastroesophageal reflux disease	1.00	(0.76–1.31)	0.98	1.00	(0.74–1.33)	0.98	0.83	(0.62–1.11)	0.21	1.00	(0.78–1.29)	0.99
Hypoxia	0.97	(0.72–1.33)	0.87	1.20	(0.88–1.64)	0.26	1.07	(0.77–1.49)	0.68	0.79	(0.59–1.05)	0.102
Pulmonary hypertension	0.87	(0.61–1.25)	0.46	1.04	(0.71–1.53)	0.83	0.83	(0.56–1.25)	0.38	0.91	(0.65–1.27)	0.57
Sleep apnea	0.84	(0.62–1.15)	0.28	0.99	(0.72–1.35)	0.93	0.76	(0.55–1.06)	0.10	1.06	(0.81–1.40)	0.66
**Medical care**												
High-resolution CT scan	1.11	(0.80–1.54)	0.52	1.07	(0.76–1.50)	0.71	1.20	(0.85–1.69)	0.30	1.05	(0.79–1.40)	0.73
Lung biopsy	1.30	(0.86–1.95)	0.21	1.03	(0.66–1.60)	0.89	1.10	(0.72–1.68)	0.66	1.23	(0.86–1.76)	0.26
Oxygen therapy or supplemental oxygen	0.90	(0.60–1.35)	0.61	1.06	(0.71–1.59)	0.77	1.11	(0.73–1.69)	0.63	1.10	(0.77–1.56)	0.60
Medication count	1.02	(0.99–1.06)	0.15	1.03	(1.00–1.07)	0.04	1.05	(1.02–1.09)	0.001	1.01	(0.99–1.04)	0.30
All spending - OOP ($1000s)	0.97	(0.92–1.03)	0.38	0.99	(0.93–1.05)	0.63	0.98	(0.92–1.04)	0.45	0.99	(0.94–1.04)	0.68
Any inpatient hospitalization	0.75	(0.51–1.10)	0.15	0.75	(0.50–1.11)	0.15	0.57	(0.38–0.85)	0.006	0.83	(0.59–1.17)	0.29
Any emergency department use	1.19	(0.85–1.65)	0.31	1.31	(0.92–1.86)	0.13	1.22	(0.87–1.71)	0.24	1.30	(0.97–1.74)	0.08
Index nintedanib prescriber was pulmonologist	0.92	(0.68–1.25)	0.59	0.88	(0.64–1.21)	0.43	0.96	(0.70–1.32)	0.82	0.91	(0.69–1.19)	0.48
Any pulmonologist visit during baseline	0.87	(0.52–1.46)	0.60	0.72	(0.42–1.24)	0.24	1.53	(0.80–2.91)	0.20	1.04	(0.63–1.73)	0.87

Notes:

1) Adjusted odds ratio and 95% CI form factional multinomial logit regression model on medication adherence trajectory groups with “high adherence” as reference group. The model specification includes an indicator for missing values for Social Deprivation Index and a constant term (not shown)

2) Sample size is 1,798 beneficiaries

3) CI: Confidence Interval

4) CT: computed tomography

5) OOP: out-of-pocket

## Discussion

Although the study of medication adherence using GBTM is not new, this study is novel in its application of the GBTM methodology to assess nintedanib adherence. This study found about 43% of patients with IPF who started nintedanib were highly adherent over one year of follow-up. In three previous studies assessing adherence by quantifying adherence as a dichotomous measure based on PDC, Investigators found adherence ranges from 51–71.3%.^15–17^ These studies used different US-based claims datasets over different time periods, which could contribute to the variation in their findings. The study most akin to ours is by Corralet al., [15] who found that 60.5% of IPF patients had at least 80% PDC for nintedanib over a 1-year period using Medicare claims data from 2014 to 2015. The analogous estimate in our study population was 65.3%.

The principal motivation for selecting GBTM to analyze adherence patterns over traditional measures such as PDC is that GBTM captures information about adherence over time, whereas traditional measures consolidate that information into a single, time-invariant quantity. That was borne out in this study; as shown in Fig. 3, only the 12% of patients in the “moderate adherence” trajectory averaged close to the estimate of 65.3% of patients with 1-year PDC ≥ 80%. Moreover, GBTM identified a third of the patient sample (“early-poor adherence” and “delayed-poor adherence” trajectories) who changed their adherence behavior less than 6 months after starting nintedanib. Although this may be explained by step-down treatment, symptom improvement or occurrence of side effects, additional research is needed to pinpoint the drivers. This change in adherence behavior over time would not have been captured by traditional measures of adherence such as PDC.

The trajectories of nintedanib adherence identified by GBTM in this study are consistent with other studies of medication adherence patterns that used the GBTM methodology. A systematic literature review of GBTM application to prescription drug adherence patterns found that most studies identified 4 to 6 trajectories, including “(a) consistent, high adherence, (b) declining adherence, (c) consistent nonadherence, and (d) initial nonadherence followed by an increase.” [21] These descriptions match well with the 5 groups identified in our study.

Some factors in this study found to be associated with higher odds of being in at least one of the poor-adherence groups were older age, female sex, and race and ethnicity other than non-Hispanic white. Comparison with the factors found to predict adherence trajectory groups in Alhazami et al.’s literature review revealed some overlap and some disagreement. In Alhazami et al., [21] racial and ethnic minority status was also associated with higher likelihood of being in a lower or declining adherence group, while the evidence on female sex predicting group membership was mixed. Younger age was also reported to be associated with poor adherence, although that might be attributable to study populations covering different age groups, as commercially insured patients are younger than fee-for-service Medicare patients. Other studies reviewed by Alhazami et al. [21] found that patients exhibiting lower socioeconomic status, higher number of comorbid conditions, and higher out-of-pocket spending also were more likely to be in poor adherence groups, where the present study did not find statistically significant associations between those predictors and group membership for nintedanib adherence.

This study also found a positive association between number of medications and odds of being in non-adherent trajectories, “high-then-poor adherence” and “delayed-poor adherence.” Taking more medications is well known to affect medication adherence of patients. [32–34] Pharmacists and clinicians can play an important role in improving medication adherence through reducing the number of medications. [35] For instance, during annual check-up visits, a comprehensive review of medications can help optimize prescription regimen. Discontinuing unnecessary and duplicate therapies, as well as combining therapies as appropriate (i.e., fixed dose combinations) can be used as regimen optimization strategies. [36].

Having at least one inpatient hospitalization was associated with lower odds of being in the “delayed-poor adherence” group. As hospitalization can be considered a proxy for disease severity, this may indicate that patients in the “moderate adherence” trajectory may have less perceived severity. Based on the health belief model, individuals who perceive their diseases as serious tend to engage in seeking treatment (e.g., being adherent to medication). [37] Patient education can be an effective strategy to make patients to fully aware of the seriousness of IPF and importance of being adherent to antifibrotics. [38].

### Strengths and limitations

We believe this study provides a nuanced understanding of nintedanib adherence among older patients with IPF. Our study is strengthened by using over 5 years of 100% Medicare data, which is critical given the comparatively high occurrence of IPF in patients older than 60 years (Raghu et al., 2018), and GBTM, a statistical methodology that enables identification of variability in medication adherence over time.

This study has some limitations. First, the use of medication claims data may not assess adherence perfectly as patients may not properly use medication after filling prescriptions. Second, claims data do not contain important measures such as disease severity and behavioral and contextual factors. Third, the accuracy of clinical information, such as IPF diagnosis, in the claims data may be low; however, this concern is assuaged by the fact that besides diagnosis requirement (one inpatient or two outpatient claims ≥ 14 days apart) implemented in our study to identify IPF patient, the prescription fill of nintedanib on the index date itself may be confirmatory as IPF is one of the FDA-approved indications of ninetdanib. Fourth, the use of Medicare data and the study sample selection criteria limit generalizability outside of older IPF patients covered by fee-for-service Medicare. Fifth, measurement of the outcome (adherence to nintedanib) does not distinguish between patients’ stopping treatment altogether versus switching to a different medication. Sixth, in summarizing adherence over a period, the GBTM approach does not incorporate any information on why adherence levels may have changed during that time. Seventh, the identified adherence trajectories are conditional on GBTM assumptions, which may not always hold. Last, the associations between GBTM-based nintedanib adherence trajectories and patient outcomes such as hospitalization or death have not yet been assessed; doing so may require addressing some methodological challenges, which need to be taken up in a later study.

## Conclusions

We used GBTM to identify five distinct trajectories of nintedanib adherence among Medicare beneficiaries with IPF. Identifying adherence trajectories and the characteristics of their members provides stakeholders with more information to personalize interventions than conventional adherence metrics. Older, female, and racial/ethnic minority patients and those with higher numbers of medications need targeted adherence intervention at specific time periods.

## Electronic supplementary material

Below is the link to the electronic supplementary material.

Supplementary Material 1

## Data Availability

The data that support the findings of this study are available from the Centers for Medicare & Medicaid Services (CMS) but restrictions apply to the availability of these data, which were used under license for the current study, and so are not publicly available. Data are however available from RESDAC@UMN.EDU upon reasonable request and with permission of the CMS-sponsored Research Assistance Data Center (ResDAC).
