# Supplementary material for: Using group based trajectory modeling for assessing medication adherence to nintedanib among idiopathic pulmonary fibrosis patients
Source: BMC Pulm Med. 2023 Jun 27;23:230. doi: 10.1186/s12890-023-02496-3 (PMC10303848; doi:10.1186/s12890-023-02496-3)

Appendix Figure 1. Mean PDC by month since initiation


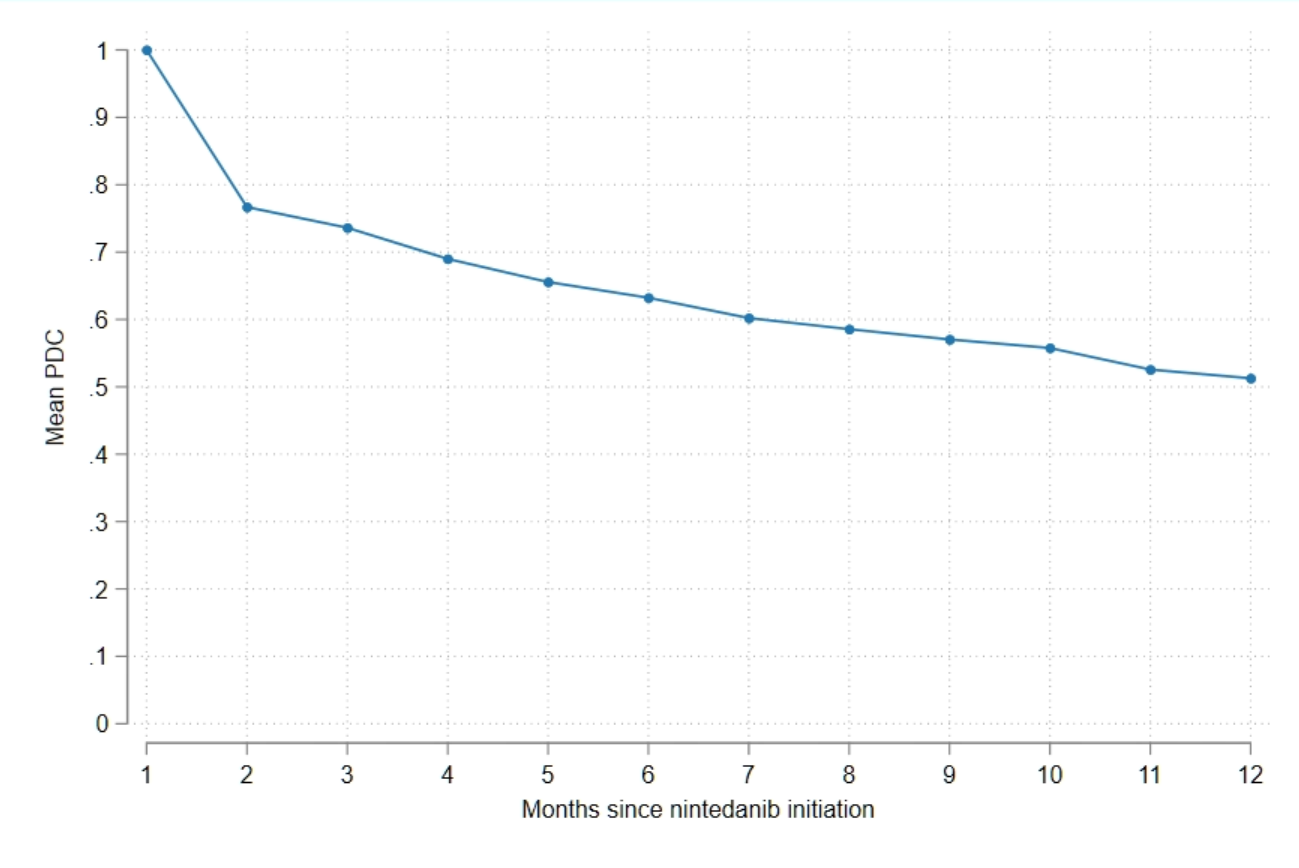


Appendix Figure 2. Spaghetti plot of individual monthly adherence trends by adherence trajectory group


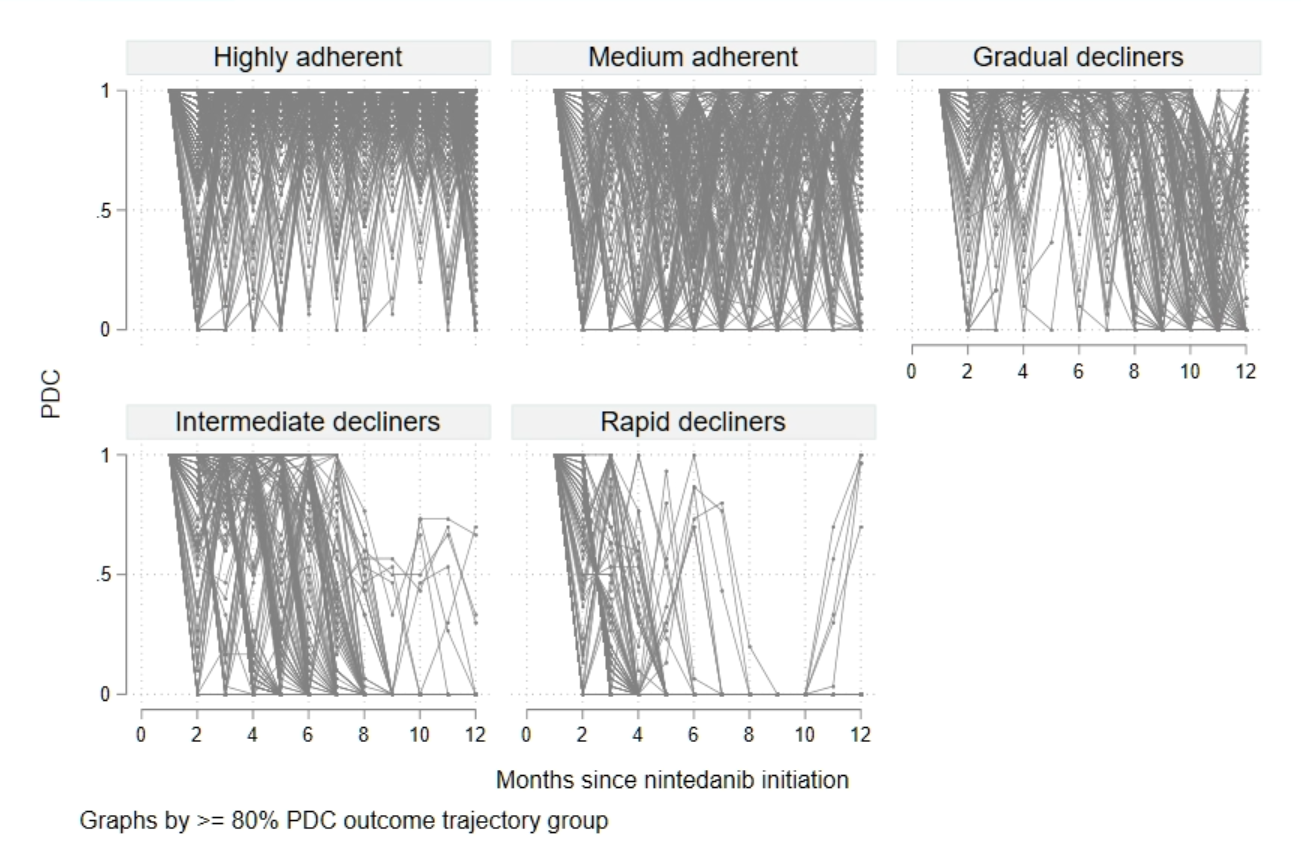

Supplement: Supplementary file 1 — Supplementary Material 1 [file 12890_2023_2496_MOESM1_ESM.docx]
